# A ‘molecular guillotine’ reveals the interphase function of Kinesin-5

**DOI:** 10.1242/jcs.210583

**Published:** 2018-02-01

**Authors:** Zhiyi Lv, Jan Rosenbaum, Timo Aspelmeier, Jörg Großhans

**Affiliations:** 1 Institute for Developmental Biochemistry, Medical School, University of Göttingen, Justus-von-Liebig Weg 11, 37077 Göttingen, Germany; 2 Institute for Mathematical Stochastics and Felix Bernstein Institute for Mathematical Statistics in the Biosciences, University of Göttingen, Goldschmidtstraße 7, 37077 Göttingen, Germany

**Keywords:** *Drosophila*, Kinesin, Molecular motor, Protease, Conditional mutant, Centrosomes, Fluctuation analysis

## Abstract

Motor proteins are important for transport and force generation in a variety of cellular processes and in morphogenesis. Here, we describe a general strategy for conditional motor mutants by inserting a protease cleavage site into the ‘neck’ between the head domain and the stalk of the motor protein, making the protein susceptible to proteolytic cleavage at the neck by the corresponding protease. To demonstrate the feasibility of this approach, we inserted the cleavage site of the tobacco etch virus (TEV) protease into the neck of the tetrameric motor Kinesin-5. Application of TEV protease led to a specific depletion and functional loss of Kinesin-5 in *Drosophila* embryos. With our approach, we revealed that Kinesin-5 stabilizes the microtubule network during interphase in syncytial embryos. The ‘molecular guillotine’ can potentially be applied to many motor proteins because Kinesins and myosins have conserved structures with accessible neck regions.

This article has an associated First Person interview with the first author of the paper.

## Abstract (precis)

**Summary:** A general strategy for conditional motor mutants that can be applied to kinesins and myosins inserts a protease cleavage site between the head and stalk domains, making it susceptible to specific proteolytic cleavage.

## INTRODUCTION

Cytoskeletal motor proteins, including myosins, dyneins and kinesins, convert the chemical energy of ATP hydrolysis into mechanical work. Motor proteins are widely involved in multiple fundamental cellular processes such as intracellular transport, cell division, cell shape change and migration (Schliwa and Woehlke, 2003). The structure of motor proteins is conserved. They contain a motor domain, referred to as the head, which catalyzes ATP and binds microtubules or F-actin. The catalytic cycle links ATP hydrolysis to a conformational change of the protein that translates into unidirectional movement of the motor protein on the filament. A second part of the protein, the stalk, links the head to the cargo binding site, contains coil-coiled structures for oligomerization or associates with other subunits. Head and stalk are parts of the same polypeptide, which is functionally relevant as a tight link of head and stalk is essential for transmission of mechanical force (Kent and Lele, 2017).

Genetic analysis of the physiological function of motor proteins has been hampered by their essential function for the cell or organism. For example, Kinesin-5 serves indispensable functions during mitosis (Heck et al., 1993), making an analysis of its function in interphase or in terminally differentiated cells difficult. Conditional mutations, such as temperature-sensitive alleles, can overcome these limitations of genetic analysis (Enos and Morris, 1990). Gene knockdown by RNAi approaches relies on protein turnover, leading to insensitivity of stable proteins. Pharmacological approaches with small-molecule inhibitors or specific antibodies provide an alternative and have been applied for motor protein inhibition (Peterson and Mitchison, 2002; Sharp et al., 2000, 1999). However, chemical approaches cannot be generalized, and need to be developed case by case.

Kinesin-5 proteins are members of kinesin superfamily, consisting of a motor domain and a coiled-coil rod with the central bipolar assembly (BASS) domain. Kinesin-5 can crosslink anti-parallel aligned microtubules by forming bipolar homo tetramers (van den Wildenberg et al., 2008). The motor activity enables filament sliding, e.g. during formation and elongation of the mitotic spindle (Kapitein et al., 2005; Shimamoto et al., 2015; Waitzman and Rice, 2014). In *Drosophila* syncytial embryos, Kinesin-5 (also known as Klp61F) is enriched at mitotic spindles (Barton et al., 1995) and is essential for spindle formation and chromosome segregation. Injection of antibodies specific for Kinesin-5 into embryos leads to a collapse of newly formed spindles and the formation of mono-asters (Sharp et al., 2000, 1999).

**Fig. 1.**
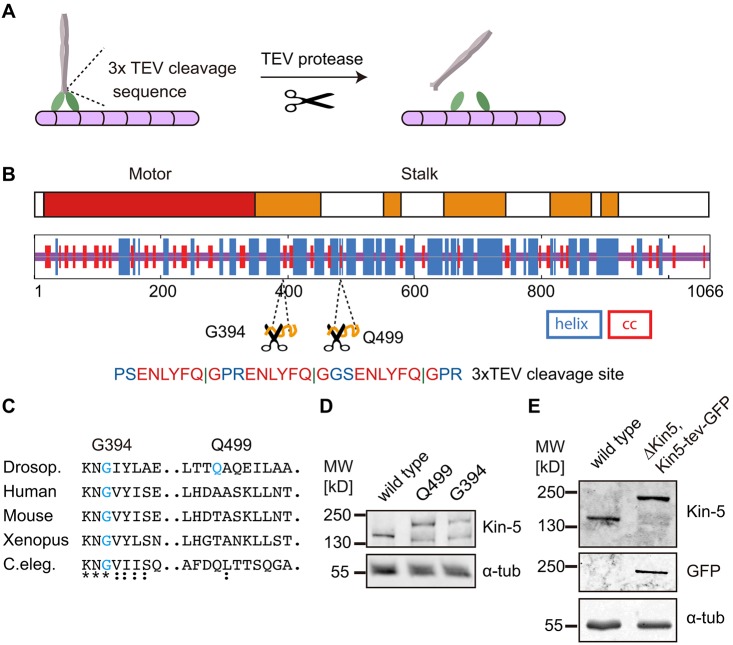
**Design of a molecular guillotine for Kinesin-5.** (A) Schematic illustration of motor protein molecular guillotine by inserting a protease substrate site next to the head domain of a motor. (B) TEV cleavage site (3×) is inserted in the coiled-coil region in the stalk domain at position G394 or Q499. Domain structure of Kinesin-5 (UniProtKB, P46863; motor domain, red; coiled-coil regions, orange) and secondary structure prediction [α-helix in blue, coiled coil (cc) in red] are indicated. (C) Sequence alignment of the insertion sites at G394 and Q499. (D,E) Western blots with embryonic extracts (0–4 h) from wild type and flies with the Kin-5[Q499tev]-GFP and Kin-5[G394tev]-GFP transgene, probed with antibodies against Kinesin-5, GFP and α-tubulin.

Making proteins susceptible to proteolytic cleavage represents a generally applicable strategy for generation of conditional alleles (Harder et al., 2008; Oliveira et al., 2010; Pauli et al., 2008). Here, we apply this concept to motor proteins by inserting a proteolytic site between the head and stalk region (the ‘neck’). We designated this strategy a ‘molecular guillotine’ (Fig. 1A). We chose the well-characterized Kinesin-5 proteins in order to demonstrate the feasibility of this approach. As a protease, we employ tobacco etch virus (TEV), which is highly specific. No match with the TEV recognition motif within the *Drosophila* proteome has been identified, and flies expressing TEV are viable and fertile (Harder et al., 2008).

## RESULTS

### Design of a ‘molecular guillotine’

We inserted three copies of the TEV recognition motif at one of two positions, G394 or Q499, into the stalk region. G394 and Q499 are located within conserved coiled-coil regions next to the head domain (Fig. 1B,C). In addition, we fused GFP to the C-terminus, which does not affect the function of Kinesin-5, as previously reported (Cheerambathur et al., 2008). These constructs were expressed as transgenes in levels comparable to the endogenous allele with a ubiquitin promoter, as assayed by western blot (Fig. 1D). As a result of the C-terminal GFP moiety, the constructs showed a slower mobility in SDS-PAGE compared with wild-type Kinesin-5. The TEV sites do not affect the functionality of Kinesin-5 as the construct with the insertion at G394 (Kin-5[G394tev]-GFP) complemented the lethality of the *Klp61f^07012^* mutation. For this, we recombined Kin-5[G394tev]-GFP with a *Klp61f* mutation. The resulting flies only expressed Kin-5[G394tev]-GFP, were viable and fertile and can be kept as a homozygous stock. In embryos from this line, Kinesin-5 was detected only at the molecular mass corresponding to transgenic Kin-5[G394tev]-GFP, which confirms the absence of endogenous Kinesin-5 (Fig. 1E).

### Kinesin-5 cleavage *in vivo*

**Fig. 2.**
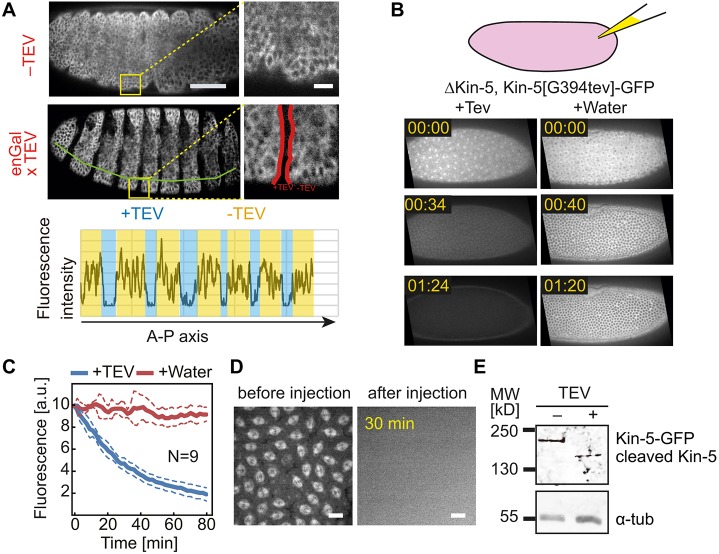
**Kin-5[G394tev]-GFP is cleaved by TEV protease.** (A) Image of living embryos expressing Kin-5[G394tev]-GFP with or without TEV protease expressed in a striped pattern. Scale bar: 50 µm. Region marked by squares in yellow are shown in high magnification on the right (scale bar: 10 µm). Quantification of GFP signal along the anterior–posterior body axis (line in green) is shown in graph below. Region of TEV expression is highlighted in red. (B–D) TEV protease or buffer was injected into syncytial embryos mutant for Kinesin-5 and expressing Kin-5[G394tev]-GFP. (B) Images from time-lapse recordings in minute:second. (C) GFP fluorescence following TEV injection. Plotted are the mean (solid) and s.d. (dashed). *N*, number of quantified embryos for each experimental condition. (D) Images of living embryos before and 30 min after injection. Scale bars: 10 µm. (E) Western blot with extracts from embryos 30 min after injection with TEV or buffer probed with Kinesin-5 and α-tubulin antibodies.

We expressed TEV protease in stripes in embryos under the control of the *engrailed* promoter. Control embryos with no TEV expression showed uniform Kin-5[G394tev]-GFP expression. In contrast, the GFP signal was strongly depleted in stripes upon expression of TEV (Fig. 2A). Next, we turned to syncytial embryos, which are characterized by their rapid and synchronous nuclear division cycles and the associated remodeling of the cytoskeleton. During mitosis, microtubules and their motors are important for the formation and function of mitotic spindles, and for chromosome segregation, whereas they function in nuclear arrangement and stabilization of the nuclear array in interphase (Kanesaki et al., 2011; Winkler et al., 2015). Kinesin-5 localizes to the mitotic spindle and is involved in chromosome segregation during mitosis (Cheerambathur et al., 2008; Sharp et al., 2000, 1999). We microinjected TEV protease into syncytial embryos and recorded GFP fluorescence. Following injection of TEV protease but not buffer, GFP fluorescence rapidly dropped (Fig. 2B). Correspondingly, the specific staining pattern, such as labeling of mitotic spindles or cytoplasmic asters was lost in TEV-injected embryos (Fig. 2D). Quantification of total GFP fluorescence provided an estimate for an approximate half life of about 30 min (Fig. 2C). Kinesin-5 was specifically cleaved, since the electrophoretic mobility of Kin-5[G394tev]-GFP was higher in TEV-injected compared with buffer-injected embryos (Fig. 2E). Kin-5[G394tev]-GFP embryos were lysed about 30 min after injection and the C-terminus of Kinesin-5 extracts analyzed by western blotting. The observed difference in electrophoretic mobility was consistent with proteolytic cleavage at the TEV sites at the neck and corresponding loss of the head domain. As we detected a single band, proteolytic cleavage was close to complete under our experimental conditions (Fig. 2E).

### Cleavage of Kinesin-5 causes the loss of function in mitosis

**Fig. 3.**
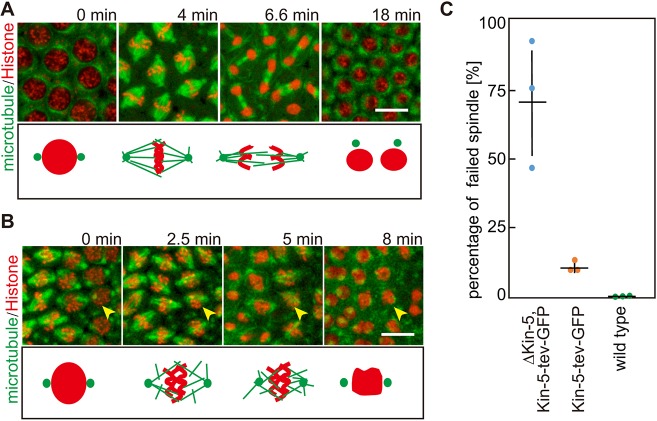
**Phenotype of Kin-5[G394tev]-GFP cleavage by TEV protease.** (A) Images from time-lapse recordings of embryos mutant for Kinesin-5 expressing the Kin-5[G394tev]-GFP, His2Av-GFP, α-Tubulin–mCherry transgene and injected with TEV protease. Arrowhead indicates embryo undergoing defective mitosis. (B) Images from the control experiment. TEV protease was injected into the embryo expressing His2Av-GFP, α-Tubulin–mCherry, without the Kin-5 guillotine. Schematic interpretation of mitotic stages are shown below each panel; red, nuclei/chromosomes; green, microtubules. Scale bars: 10 µm. (C) Proportion of abnormal spindles after TEV injection. Data are mean±s.d. *N*=148 spindles in 3 embryos for ΔKin-5, Kin-5-tev-GFP, 348 spindles in 3 embryos for Kin-5-tev-GFP and 1384 spindles in 3 embryos for wild type. Source data are listed in Table S1.

Next, we analyzed the functional consequences of Kinesin-5 cleavage. To track the nuclear cycles and behavior of chromosomes, we co-injected fluorescently labeled histone-1 and TEV protease into *Klp61f* null embryos expressing the Kin-5[G394tev]-GFP transgene (ΔKin5, Kin-5-tev-GFP). Following TEV injection, we observed a failure of chromosome separation and monoastral spindles (Fig. 3A,B). These phenotypes were observed in individual spindles interspersed between normally appearing spindles. These phenotypes were consistent with the previously reported mitotic defects following injection of antibody against Kinesin-5 (Sharp et al., 1999). We quantified the percentage of failed spindles in the following embryos after TEV injection: (1) ΔKin-5, Kin-5-tev-GFP, (2) Kin-5-tev-GFP and (3) wild type (Fig. 3C). Whereas no defects were observed in wild-type embryos, about three-quarters of the spindles did not form or collapsed in embryos containing only the TEV-cleavable Kinesin-5 protein. The proportion of abnormal spindles was ∼10%, if both TEV-cleavable and wild-type Kinesin-5 was present. This indicates that the TEV-cleavable form of Kinesin-5 exerts a dominant-negative activity on the wild-type protein, consistent with Kinesin-5 acting as a homo-tetramer.

### Interphase function of Kinesin-5 in syncytial embryos

**Fig. 4.**
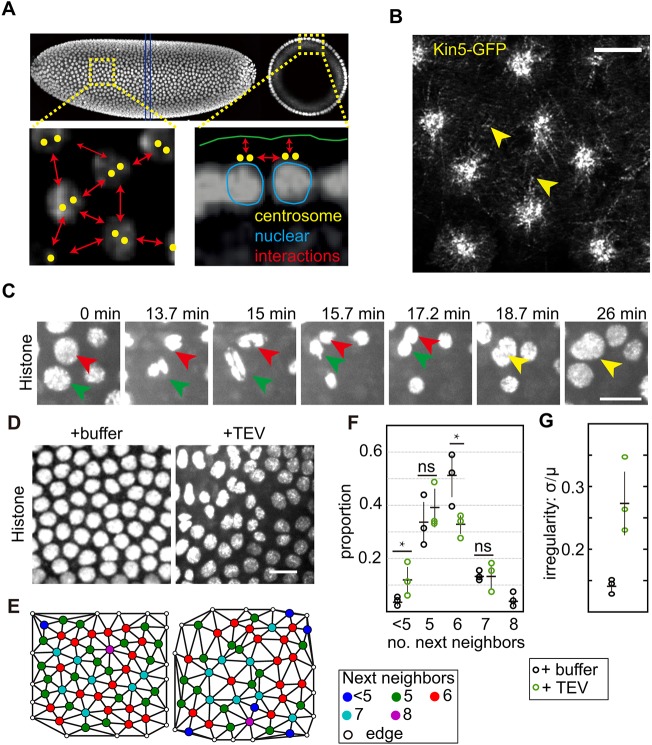
**Kinesin-5 is important for nuclear positioning in interphase.** (A) Projected image of an embryo expressing Histone 2Av from selective plane illumination microscopy in side view and cross section (position indicated by lines in blue). Magnified sections illustrate the interactions between the nuclei and between nuclei and cortex, respectively. Dots in yellow indicate centrosome pairs. (B) Image of living embryo expressing Kin-5–GFP (apical position) Scale bar: 5 µm. (C) Images from time-lapse recording of embryos mutant for Kinesin-5 expressing the Kin-5[G394tev]-GFP transgene and co-injected with TEV protease and fluorescent labeled Histone H1. The green and red arrowheads indicate two nuclei that underwent a nuclear division cycle. After successful daughter nuclei separation, the two non-daughter nuclei fuse as indicated by yellow arrowhead. Scale bar: 10 µm. (D) Nuclei labeled with injected Histone H1. Embryos (Kinesin-5 mutant with Kin-5[G394tev]-GFP transgene) injected with buffer or TEV protease and fluorescently labeled Histone H1 to label nuclei. Scale bar: 10 µm. (E) Segmented images from D. Color coding for the number of neighboring cells is indicated. The nuclei labeled with empty circles were not included in the calculation. (F) Proportion of nuclei according to number of neighbors and (G) irregularity of nuclear arrangement in embryos injected with buffer or TEV protease. The irregularity parameter σ/μ in Kinesin-5-depleted and control embryo. Data are mean±s.d. *N*=105 nuclei in 3 embryos for TEV injection and 152 nuclei in 3 embryos for buffer injection. Statistical significance calculated by Student's *t*-test; *P<0.05; ns, not significant. Source data are listed in Table S1.

Compared with the well-established mitotic function, much less is known about how Kinesin-5 is involved in microtubule organization and function in interphase. In interphases of syncytial embryos, nuclei and the associated centrosomes form a regular arrangement (Fig. 4A), and Kinesin-5–GFP is strongly enriched at the centrosomes and associated asters. In addition, dynamic extended structures between adjacent asters were detected (Fig. 4B). These transient signals may represent microtubules coated with Kinesin-5 and possibly antiparallel aligned microtubules. We noticed that in Kinesin-5-depleted embryos, two nuclei originating from different spindles fused together after successful separation from their own sister nuclei (Fig. 4C), which implies an interphase function of Kinesin-5 for keeping nuclei separate and at a distance. As hypothesized previously (Kanesaki et al., 2011; Winkler et al., 2015), Kinesin-5 may be involved in nuclear positioning and formation of the nuclear array in syncytial *Drosophila* embryos. We examined the position of the nuclei in interphase carefully (Fig. 4D,E) and quantified the number of neighboring cells for each nucleus (Fig. 4F). In control embryos, 50% of nuclei had 6 neighbors, whereas 33% and 13% had 5 and 7 neighbors, respectively. In contrast, the distribution was different in the Kinesin-5-depleted embryos, with significantly fewer nuclei with 6 neighbors and significantly more with fewer than 5, or more than 7 neighbors (Fig. 4F). As a measure of the regularity of the nuclear arrangement, we calculated the variation in distances to neighboring cells [standard deviation (σ) normalized to the distance average (µ)]. We detected a much higher variation in Kinesin-5-depleted embryos compared with the control embryo (Fig. 4G). In summary, our data indicate that Kinesin-5 is involved in interactions between adjacent asters leading to a normal arrangement of the nuclei in interphase.

**Fig. 5.**
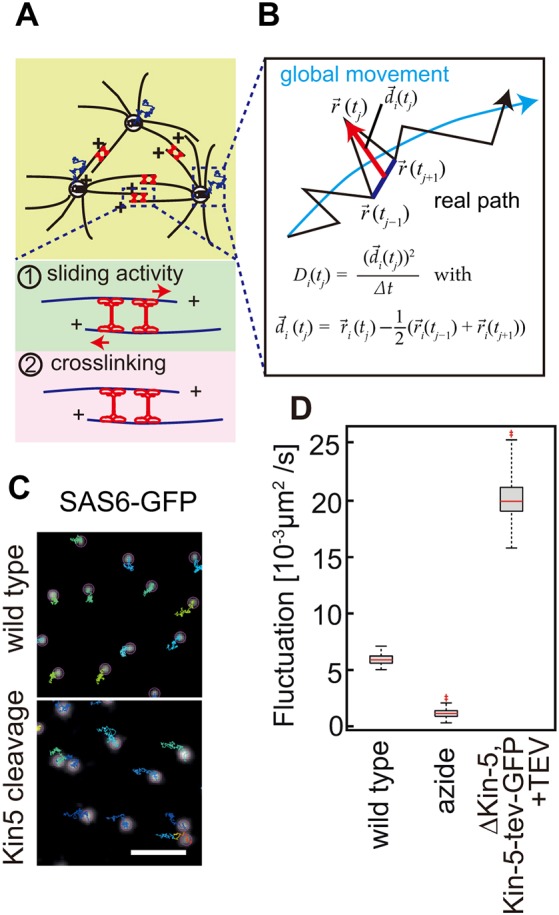
**Kinesin-5 suppresses centrosome fluctuation in interphase.** (A) Illustration of microtubule asters with overlapping microtubules in anti-parallel orientation. Kinesin-5 may slide microtubules apart (1) or crosslink adjacent asters (2). (B) Definition of the fluctuation parameter *D* as the deviation from slower ‘drift’ movement. The fluctuation parameter has the dimension of a diffusion constant and can be regarded as apparent diffusion. (C) Images from living embryo mutant for Kinesin-5 expressing Kin-5[G394tev]-GFP and SAS6-GFP and injection with TEV protease or buffer. Trajectories of centrosomes over 220 s on an image from time-lapse recording. Scale bar: 5 µm. (D) Box plot displaying time-averaged fluctuation of centrosomes in embryos expressing SAS-6-GFP injected with buffer (wild type, 5 embryos, 1757 centrosomes), sodium azide (2 embryos, 228 centrosomes) or TEV protease for cleavage of Kin-5[G394tev]-GFP (2 embryos, 660 centrosomes). The bottom and top of the box are the first and third quartiles, band inside the box is the median.Whiskers mark the minimum and maximum of the data set. ‡*P*<0.05, Student's *t*-test Source data are listed in Table S1.

Kinesin-5 bound to anti-parallel aligned microtubules may push adjacent asters away from each other and thus generate a repulsive force, which may lead to uniform internuclear distances. In this model, Kinesin-5 would promote movements of centrosome and their associated asters. Alternatively, Kinesin-5 may crosslink microtubules from adjacent asters and stabilize the syncytial microtubule network. In this alternative model Kinesin-5 would suppress movement of centrosomes and associated asters (Fig. 5A). To distinguish these two models, we recorded the dynamics of centrosomes in the scale of seconds and calculated second-scale fluctuations of the centrosomes defined as previously reported (Fig. 5B,C) (Winkler et al., 2015). We calculated fluctuations, *D_i_*(*t_j_*), as the deviation from slow (minute-scale) drift movements averaged over time and multiple centrosomes. These fluctuations have the dimension of a diffusion constant and do not contain slow drift movements. Comparing buffer- and TEV-injected embryos, we found that the fluctuations were strongly increased (about threefold) in Kinesin-5-depleted embryos. The baseline for passive, non-ATP-dependent fluctuations is about five times below that of the wild-type (Winkler et al., 2015) (Fig. 5D). Owing to the increased mobility of centrosomes in Kinesin-5-depleted embryos, we conclude that Kinesin-5 functions in the stabilization of the array of microtubule asters by crosslinking, for example.

## DISCUSSION

The function of Kinesin-5 in spindle formation and elongation during mitosis is well established (Heck et al., 1993; Sharp et al., 2000). Consistently, depletion of Kinesin-5 by our guillotine method induced defects in chromosome segregation in syncytial *Drosophila* embryos consistent with previous reports (Sharp et al., 1999). Aside from its role in mitotic spindle formation, our insights are limited into the functions of Kinesin-5 during interphase. Study of interphase is hampered by the difficulty that interphase functions are obscured by mitotic defects. In the case of *Drosophila*, embryos depleted of Kinesin-5 cannot be obtained, since Kinesin-5 has an essential function during oogenesis (Radford et al., 2017). By employing small-molecule inhibitors, Kinesin-5 was found to be involved in maintenance of neuron dendritic structure (Kahn et al., 2015) and intracellular transport from Golgi to the cell surface in cultured cells (Wakana et al., 2013). As another approach for conditional interference, we developed a method for decapitating motor proteins by proteolysis or the ‘molecular guillotine’. This method is potentially suitable for many motor proteins. We demonstrate the feasibility of this approach with Kinesin-5 and TEV protease. We revealed an interphase function of Kinesin-5 in syncytial *Drosophila* embryos for the stabilization of the microtubule network and keeping adjacent nuclei at a distance.

In syncytial embryos, the microtubule asters originating from centrosomes can directly interact with neighboring asters, since they are not physically separated by plasma membranes. These interactions lead to the formation of an extended network covering the embryonic cortex. The phenotypic behavior of centrosomes and their associated nuclei reflect their intrinsic properties but also, as part of the network, include any influences from the neighbors. Adjacent microtubule asters potentially interact via crosslinkers such as Feo/Ase1p, bundling proteins or motors with sliding activity, such as Kinesin-5. Here, we tested the hypothesis that Kinesin-5 generates repulsive forces between adjacent astral microtubules in interphase. We expected that a loss of force generation would lead to a reduced mobility of the network and its nodes, the centrosomes. Using the fluctuations of centrosomes as an indicator of network dynamics, we rejected our hypothesis, because we measured an increased mobility of the centrosomes, when Kinesin-5 was inactivated. These data suggest that the function of Kinesin-5 as a crosslinker is more dominant *in vivo* than its function for sliding of anti-parallel aligned microtubules and thus pushing apart adjacent microtubule asters. The *in vivo* function of Kinesin-5 is similar to that of kinesin-1, which is enriched at the cortex and in F-actin and actin caps (Winkler et al., 2015). Both may be involved in anchoring microtubule asters to the cortex and in this way counteract the fluctuation movements of centrosomes. Having identified a stabilizing function of Kinesin-5, the question remains about the origin of the forces driving centrosome fluctuations. Fluctuations are due to an active component, since ATP depletion leads to loss of fluctuations. The minus-end directed motor kinesin-14 may serve as a force generator (Sharp et al., 2000).

The ‘molecular guillotine’ is potentially a versatile method for conditional inactivation of motor proteins. TEV protease has been employed for inactivation of cohesin in yeast (Uhlmann et al., 2000) and fly (Pauli et al., 2008), as well as for inactivation of claudin in *Drosophila* (Harder et al., 2008). However, this approach had not been applied for inactivation of motor proteins to our knowledge. The approach of a ‘molecular guillotine’ as reported in this study can be applied widely to members of the motor protein families. Unlike using the small-molecule inhibitor (Engelke et al., 2016; Peterson and Mitchison, 2002), TEV protease can be specifically and conditionally expressed using the UAS-GAL4 system and its temperature-dependent variants in any genetically tractable cell type. This allows ‘decapitation’ of the selected motor protein in a tissue- and developmental stage-specific manner. The molecular guillotine offers an alternative approach to other previously reported conditional approaches based on protein degradation such as the auxin-inducible degradation system (Gray et al., 2001), the degron (Dohmen et al., 1994) or deGradFP systems (Caussinus et al., 2012), which rely on the ubiquitin-mediated protein degradation machinery. In the case of TEV cleavage, a single peptide bond is cleaved, whereas in the degron or deGradFP systems, the protein complex is subjected to complete proteolysis in the proteasome. Direct comparison of these systems will reveal the respective advantages and disadvantages with respect to kinetics and effectiveness of motor depletion. A second intrinsic difference to degradation-based systems is a potential dominant effect of the molecular guillotine. The single cut generates two parts, which may interact with uncut counterparts and thus induce a dominant effect. We detected evidence for such a dominant effect, when the phenotype caused by addition of TEV protease is also detected in heterozygous embryos containing a Kinesin-5 wild-type allele and a copy of the Kin-5-tev-GFP transgene.

In summary, the novel approach of a molecular guillotine enabled us to investigate a specific function of the motor protein Kinesin-5 in interphase. Potentially, the decapitation approach can be correspondingly applied to other kinesin motors, as well as myosins, as they have a related domain structure in common.

## MATERIALS AND METHODS

### Genetics

Fly stocks (en-Gal4, α-Tubulin-mCherry, His2Av-GFP, Sas6-GFP, Klp61f^07012^) (Peel et al., 2007; Spradling et al., 1999) were obtained from the Bloomington Stock Center, if not otherwise noted, and genetic markers and annotations are described in FlyBase (Gramates et al., 2017). Transgenes of ubi-Kin5[Q499tev]-GFP, ubi-Kin5[G394tev]-GFP and sqh-Kin5-GFP were generated by P-element-mediated random genome integration. We isolated multiple insertions on the third chromosome with varying expression. The ubi-Kin5-tev-GFPG394 line with strongest GFP fluorescence was recombined with the amorphic *Klp61f^07012^* mutation and kept as a homozygous line. Similarly, transgenes of spq-Kin5–GFP [expression driven by the Spaghetti Squash (*spq*) promoter] without TEV sites complemented the lethality of *Klp61f^07012^* and were kept as homozygous stocks. TEV protease was expressed from a UASt-TEV transgene (Harder et al., 2008) or injected as a purified recombinant protein.

### Cloning

A sequence coding for three recognition sites of TEV protease (PS ENLYFQG PR ENLYFQG GS ENLYFQG PR) was inserted behind the codons of G394 or Q499 of the *Klp61f* cDNA (SK-Klp61f, *Drosophila* Genomic Resource Center, Bloomington). The resulting coding sequence (Kinesin-tev) was cloned behind the ubiquitin promoter (Lee et al., 1988; Oda and Tsukita, 2001) and 5′ to eGFP into the multiple cloning site of a pUASt vector derivative lacking the UAS and hsp70TATA sites. The Kinesin-5–GFP fusion constructs were generated by cloning the *Klp61f* cDNA (from SK-klp61f) with GFP inserted at the C-terminus into the transformation vector sGMCA (Kiehart et al., 2000), which contains the *spq* promoter for ubiquitous expression. Sequence information and details of the cloning procedure are available upon request.

### Western blotting

The *Klp61f* coding sequence corresponding to the C-terminal tail (aa 600–1066) was cloned by PCR with SK-Klp61f (*Drosophila* Genomic Resource Center, Bloomington) as template into a protein expression vector with a N-terminal 9×His tag. The His9-Kinesin-5-C600 protein with an apparent molecular mass of ∼70 kDa in SDS polyacrylamide gel electrophoresis (SDS-PAGE) was purified under denaturing conditions (Trenzyme, Konstanz) and used for immunization of rabbits (BioGenes, Berlin). Embryonic extracts were analyzed by SDS-PAGE and immunoblotting as previously described (Wenzl et al., 2010). Briefly, proteins were blotted to nitrocellulose filters by wet transfer (100 mA per mini gel, overnight). The blots were blocked with 5% fat-free milk in phosphate buffered saline (PBS), incubated with the following primary antibodies in PBT (PBS with 0.1% Tween-20), rabbit anti-Kinesin-5 (1:5000, this study), mouse anti-tubulin (1:100,000, B512, Sigma, T5168), rabbit anti-GFP (1:5000, Torrey Pines Biolabs, TP401) and fluorescently labeled secondary antibodies (LiCOR, 1:20,000; 0.05 µg/ml in PBT) for each 2 h at room temperature. The developed blots were imaged with a LICOR system.

### Microinjection

Embryos were dechorionated and aligned on a coverslip, desiccated for 10 min, and covered with halocarbon oil (Voltalef 10S, Lehmann & Voss). We injected TEV protease at 10 µM purified from overexpressing *E. coli* (a gift from Dirk Görlich, Max Planck Institute, Göttingen, Germany). Histone-1 Alexa Fluor 488-conjugated protein (ThermoFisher) was injected at a concentration of 2 mg/ml.

### Microscopy

Images were recorded with a Zeiss microscope equipped with a spinning disc (25×/NA0.7 multi immersion, 40×/NA1.3 oil). Centrosome movement was recorded in Sas6–GFP-expressing embryos as previously described with a frame rate of 1 Hz (Winkler et al., 2015). Kinesin-5–GFP distribution in interphase was recorded with a confocal microscope (Zeiss LSM780 with airy scan unit, 63×NA 1.4/oil). Images were processed with Fiji/ImageJ (Schindelin et al., 2012).

### Fluctuation analysis

Centrosome tracking and measurement of fluctuation were carried out as previously described (Winkler et al., 2015). The custom-made software code is available on request.

## Supplementary Material

Supplementary information

## References

[JCS210583C1] Barton N. R., Pereira A. J. and Goldstein L. S. (1995). Motor activity and mitotic spindle localization of the Drosophila kinesin-like protein KLP61F. *Mol. Biol. Cell* 6, 1563-1574. 10.1091/mbc.6.11.1563 8589456 PMC301311

[JCS210583C2] Caussinus E., Kanca O. and Affolter M. (2012). Fluorescent fusion protein knockout mediated by anti-GFP nanobody. *Nat. Struct. Mol. Biol.* 19, 117-121. 10.1038/nsmb.2180 22157958

[JCS210583C3] Cheerambathur D. K., Brust-Mascher I., Civelekoglu-Scholey G. and Scholey J. M. (2008). Dynamic partitioning of mitotic Kinesin-5 cross-linkers between microtubule-bound and freely diffusing states. *J. Cell Biol.* 182, 429-436. 10.1083/jcb.200804100 18678711 PMC2500124

[JCS210583C4] Dohmen R. J., Wu P. and Varshavsky A. (1994). Heat-inducible degron: a method for constructing temperature-sensitive mutants. *Science* 263, 1273-1276. 10.1126/science.8122109 8122109

[JCS210583C5] Engelke M. F., Winding M., Yue Y., Shastry S., Teloni F., Reddy S., Blasius T. L., Soppina P., Hancock W. O., Gelfand V. I. et al. (2016). Engineered kinesin motor proteins amenable to small-molecule inhibition. *Nat. Commun.* 7, 11159 10.1038/ncomms11159 27045608 PMC4822052

[JCS210583C6] Enos A. P. and Morris N. R. (1990). Mutation of a gene that encodes a kinesin-like protein blocks nuclear division in A. nidulans. *Cell* 60, 1019-1027. 10.1016/0092-8674(90)90350-N 2138511

[JCS210583C7] Gramates L. S., Marygold S. J., Santos G. D., Urbano J.-M., Antonazzo G., Matthews B. B., Rey A. J., Tabone C. J., Crosby M. A., Emmert D. B. et al. (2017). FlyBase at 25: looking to the future. *Nucleic Acids Res.* 45, D663-D671. 10.1093/nar/gkw1016 27799470 PMC5210523

[JCS210583C8] Gray W. M., Kepinski S., Rouse D., Leyser O. and Estelle M. (2001). Auxin regulates SCF(TIR1)-dependent degradation of AUX/IAA proteins. *Nature* 414, 271-276. 10.1038/35104500 11713520

[JCS210583C34] Harder B., Schomburg A., Pflanz R., Küstner K., Gerlach N. and Schuh R. (2008). TEV protease-mediated cleavage in Drosophila as a tool to analyze protein functions in living organisms. *Biotechniques* 44, 765-772. 10.2144/000112884 18476830

[JCS210583C9] Heck M. M., Pereira A., Pesavento P., Yannoni Y., Spradling A. C. and Goldstein L. S. (1993). The kinesin-like protein KLP61F is essential for mitosis in Drosophila. *J. Cell Biol.* 123, 665-679. 10.1083/jcb.123.3.665 8227131 PMC2200134

[JCS210583C10] Kahn O. I., Sharma V., González-Billault C. and Baas P. W. (2015). Effects of Kinesin-5 inhibition on dendritic architecture and microtubule organization. *Mol. Biol. Cell* 26, 66-77. 10.1091/mbc.E14-08-1313 25355946 PMC4279230

[JCS210583C11] Kanesaki T., Edwards C. M., Schwarz U. S. and Grosshans J. (2011). Dynamic ordering of nuclei in syncytial embryos: a quantitative analysis of the role of cytoskeletal networks. *Integr. Biol.* 3, 1112-1119. 10.1039/c1ib00059d 22001900

[JCS210583C12] Kapitein L. C., Peterman E. J. G., Kwok B. H., Kim J. H., Kapoor T. M. and Schmidt C. F. (2005). The bipolar mitotic kinesin Eg5 moves on both microtubules that it crosslinks. *Nature* 435, 114-118. 10.1038/nature03503 15875026

[JCS210583C13] Kent I. A. and Lele T. P. (2017). Microtubule-based force generation. *Wiley Interdiscip. Rev. Nanomed. Nanobiotechnol.* 9, e1428 10.1002/wnan.1428 PMC5326615 27562344

[JCS210583C14] Kiehart D. P., Galbraith C. G., Edwards K. A., Rickoll W. L. and Montague R. A. (2000). Multiple forces contribute to cell sheet morphogenesis for dorsal closure in Drosophila. *J. Cell Biol.* 149, 471-490. 10.1083/jcb.149.2.471 10769037 PMC2175161

[JCS210583C15] Lee H. S., Simon J. A. and Lis J. T. (1988). Structure and expression of ubiquitin genes of Drosophila melanogaster. *Mol. Cell. Biol.* 8, 4727-4735. 10.1128/MCB.8.11.4727 2463465 PMC365564

[JCS210583C16] Oda H. and Tsukita S. (2001). Real-time imaging of cell-cell adherens junctions reveals that Drosophila mesoderm invagination begins with two phases of apical constriction of cells. *J. Cell Sci.* 114, 493-501. 11171319 10.1242/jcs.114.3.493

[JCS210583C17] Oliveira R. A., Hamilton R. S., Pauli A., Davis I. and Nasmyth K. (2010). Cohesin cleavage and Cdk inhibition trigger formation of daughter nuclei. *Nat. Cell Biol.* 12, 185-192. 10.1038/ncb2018 20081838 PMC3284228

[JCS210583C18] Pauli A., Althoff F., Oliveira R. A., Heidmann S., Schuldiner O., Lehner C. F., Dickson B. J. and Nasmyth K. (2008). Cell-type-specific TEV protease cleavage reveals cohesin functions in Drosophila neurons. *Dev. Cell* 14, 239-251. 10.1016/j.devcel.2007.12.009 18267092 PMC2258333

[JCS210583C19] Peel N., Stevens N. R., Basto R. and Raff J. W. (2007). Overexpressing centriole-replication proteins in vivo induces centriole overduplication and de novo formation. *Curr. Biol.* 17, 834-843. 10.1016/j.cub.2007.04.036 17475495 PMC1885955

[JCS210583C20] Peterson J. R. and Mitchison T. J. (2002). Small molecules, big impact: a history of chemical inhibitors and the cytoskeleton. *Chem. Biol.* 9, 1275-1285. 10.1016/S1074-5521(02)00284-3 12498880

[JCS210583C21] Radford S. J., Go A. M. M. and McKim K. S. (2017). Cooperation between Kinesin motors promotes spindle symmetry and chromosome organization in oocytes. *Genetics* 205, 517-527. 10.1534/genetics.116.194647 27932541 PMC5289833

[JCS210583C22] Schindelin J., Arganda-Carreras I., Frise E., Kaynig V., Longair M., Pietzsch T., Preibisch S., Rueden C., Saalfeld S., Schmid B. et al. (2012). Fiji: an open-source platform for biological-image analysis. *Nat. Methods* 9, 676-682. 10.1038/nmeth.2019 22743772 PMC3855844

[JCS210583C23] Schliwa M. and Woehlke G. (2003). Molecular motors. *Nature* 422, 759-765. 10.1038/nature01601 12700770

[JCS210583C24] Sharp D. J., Yu K. R., Sisson J. C., Sullivan W. and Scholey J. M. (1999). Antagonistic microtubule-sliding motors position mitotic centrosomes in Drosophila early embryos. *Nat. Cell Biol.* 1, 51-54. 10.1038/9025 10559864

[JCS210583C25] Sharp D. J., Brown H. M., Kwon M., Rogers G. C., Holland G. and Scholey J. M. (2000). Functional coordination of three mitotic motors in Drosophila embryos. *Mol. Biol. Cell* 11, 241-253. 10.1091/mbc.11.1.241 10637305 PMC14771

[JCS210583C26] Shimamoto Y., Forth S. and Kapoor T. M. (2015). Measuring pushing and braking forces generated by ensembles of Kinesin-5 crosslinking two microtubules. *Dev. Cell* 34, 669-681. 10.1016/j.devcel.2015.08.017 26418296 PMC4604754

[JCS210583C27] Spradling A. C., Stern D., Beaton A., Rhem E. J., Laverty T., Mozden N., Misra S. and Rubin G. M. (1999). The Berkeley Drosophila Genome Project gene disruption project: single P-element insertions mutating 25% of vital Drosophila genes. *Genetics* 153, 135-177. 10471706 10.1093/genetics/153.1.135 PMC1460730

[JCS210583C28] Uhlmann F., Wernic D., Poupart M.-A., Koonin E. V. and Nasmyth K. (2000). Cleavage of cohesin by the CD clan protease separin triggers anaphase in yeast. *Cell* 103, 375-386. 10.1016/S0092-8674(00)00130-6 11081625

[JCS210583C29] van den Wildenberg S. M. J. L., Tao L., Kapitein L. C., Schmidt C. F., Scholey J. M. and Peterman E. J. G. (2008). The homotetrameric Kinesin-5 KLP61F preferentially crosslinks microtubules into antiparallel orientations. *Curr. Biol.* 18, 1860-1864. 10.1016/j.cub.2008.10.026 19062285 PMC2657206

[JCS210583C30] Waitzman J. S. and Rice S. E. (2014). Mechanism and regulation of Kinesin-5, an essential motor for the mitotic spindle. *Biol. Cell* 106, 1-12. 10.1111/boc.201300054 24125467 PMC4034379

[JCS210583C31] Wakana Y., Villeneuve J., van Galen J., Cruz-Garcia D., Tagaya M. and Malhotra V. (2013). Kinesin-5/Eg5 is important for transport of CARTS from the trans-Golgi network to the cell surface. *J. Cell Biol.* 202, 241-250. 10.1083/jcb.201303163 23857769 PMC3718972

[JCS210583C32] Wenzl C., Yan S., Laupsien P. and Großhans J. (2010). Localization of RhoGEF2 during Drosophila cellularization is developmentally controlled by slam. *Mech. Dev.* 127, 371-384. 10.1016/j.mod.2010.01.001 20060902

[JCS210583C33] Winkler F., Gummalla M., Künneke L., Lv Z., Zippelius A., Aspelmeier T. and Grosshans J. (2015). Analysis of Centrosomes Reveals a Cortical Function of Kinesin-1. *Biophys. J.* 109, 856-868. 10.1016/j.bpj.2015.07.044 26331244 PMC4564942

