# Supplementary material for: A ‘molecular guillotine’ reveals the interphase function of Kinesin-5
Source: J Cell Sci. 2018 Feb 1;131(3):jcs210583. doi: 10.1242/jcs.210583 (PMC5826049; doi:10.1242/jcs.210583)
Supplement: Supplementary information [file joces-131-210583-s1.pdf]

Table S1. Source data relating to results shown in Figs 1-5.

Click here to Download Table S1

Journal of Cell Science • Supplementary information
